# The Role of the Loading Condition in Predictions of Bone Adaptation in a Mouse Tibial Loading Model

**DOI:** 10.3389/fbioe.2021.676867

**Published:** 2021-06-11

**Authors:** Vee San Cheong, Visakan Kadirkamanathan, Enrico Dall’Ara

**Affiliations:** ^1^Insigneo Institute for in Silico Medicine, University of Sheffield, Sheffield, United Kingdom; ^2^Department of Automatic Control and Systems Engineering, University of Sheffield, Sheffield, United Kingdom; ^3^Department of Oncology and Metabolism, University of Sheffield, Sheffield, United Kingdom

**Keywords:** micro-FE, bone remodeling, micro CT analysis, longitudinal imaging study, mechanical loading effect

## Abstract

The *in vivo* mouse tibial loading model is used to evaluate the effectiveness of mechanical loading treatment against skeletal diseases. Although studies have correlated bone adaptation with the induced mechanical stimulus, predictions of bone remodeling remained poor, and the interaction between external and physiological loading in engendering bone changes have not been determined. The aim of this study was to determine the effect of passive mechanical loading on the strain distribution in the mouse tibia and its predictions of bone adaptation. Longitudinal micro-computed tomography (micro-CT) imaging was performed over 2 weeks of cyclic loading from weeks 18 to 22 of age, to quantify the shape change, remodeling, and changes in densitometric properties. Micro-CT based finite element analysis coupled with an optimization algorithm for bone remodeling was used to predict bone adaptation under physiological loads, nominal 12N axial load and combined nominal 12N axial load superimposed to the physiological load. The results showed that despite large differences in the strain energy density magnitudes and distributions across the tibial length, the overall accuracy of the model and the spatial match were similar for all evaluated loading conditions. Predictions of densitometric properties were most similar to the experimental data for combined loading, followed closely by physiological loading conditions, despite no significant difference between these two predicted groups. However, all predicted densitometric properties were significantly different for the 12N and the combined loading conditions. The results suggest that computational modeling of bone’s adaptive response to passive mechanical loading should include the contribution of daily physiological load.

## Introduction

Bone is a dynamic tissue that adapts its mass and geometry to mechanical and biological factors. Its adaptive nature is governed by processes of modeling and remodeling in response to the stimulus (Kim et al., 2003; Meakin et al., 2017), collectively known as bone (re)modeling or bone adaptation. Bone adaptation is key for bone homeostasis (Javaheri et al., 2020). Age and diseases such as osteoporosis disrupt this balance by causing a net bone loss and deterioration in mechanical properties (Birkhold et al., 2014; Razi et al., 2015b; Meakin et al., 2017; Roberts et al., 2019).

Preclinical models are useful in elucidating the mechanisms behind the regulation of bone adaptation, and the mouse tibial axial compression loading model is frequently used due to the non-invasive application of loads through the knee and ankle joints (Main et al., 2020). By controlling the applied axial load on the tibia, this animal model has the goal of increasing the local deformation of the bone tissue, similar to what happens in impact exercises (De Souza et al., 2005; Main et al., 2020). Cross-sectional and longitudinal studies have demonstrated that increased passive loading on the skeleton is effective at inducing increased bone formation in aged (Birkhold et al., 2014; Razi et al., 2015a, b) and ovariectomized (Roberts et al., 2019) mouse tibiae, albeit with lower adaptive response than in healthy mice (Melville et al., 2014). *In vivo* imaging and dynamic 4D (time and space) assessment of bone adaptation enable the detailed evaluation of the lasting benefits of mechanical loading on healthy (Javaheri et al., 2020) and ovariectomized (Roberts et al., 2020) mouse tibia during treatment and after its withdrawal. An understanding of how mechanical loading modifies baseline bone adaptation in response to normal physiological loading will benefit the optimization of treatment strategies to arrest bone loss, improve fracture healing and enhance rehabilitation (Cheong et al., 2020a; Main et al., 2020).

The coupling of experimental studies with computational models has enabled the processes governing the mechanical regulation of bone adaptation to be explored (Schulte et al., 2013a; Cheong et al., 2020b; Javaheri et al., 2020). Bone adaptation measured from the comparison of *in vivo* micro-CT images or endpoint histology is correlated with the mechanical environment computed from micro-CT based micro finite element (micro-FE) models, to determine the role of the mechanical stimulus in enhancing bone formation (Webster et al., 2012; Carriero et al., 2018). The challenge is to replicate the bone adaptation in response to passive mechanical loading. Most studies have simulated the nominal condition imposed during the tibial loading experiment by fully constraining the distal end, and applying a pure axial load to the proximal end (Yang et al., 2014; Pereira et al., 2015; Razi et al., 2015b), after alignment of the bone to the experimental loading configuration. Bone remodeling algorithms can then be coupled to the local mechanical stimuli to better understand the process of bone adaptation. Several categories of bone remodeling algorithms have been proposed, including those based on a global optimality criterion (Jang and Kim, 2010), achieving a state of homeostasis (Schulte et al., 2013b), damage repair (Hambli, 2014), and mechano-chemo-biological models (Lerebours et al., 2016). In the mouse model, predictions of bone adaptation have largely centered on attaining homeostasis, based on Frost’s mechanostat (Schulte et al., 2013b; Levchuk et al., 2014; Pereira et al., 2015; Carriero et al., 2018). Nevertheless, no study has analyzed the contribution of physiological loading in understanding the response of passive mechanical loading, with studies applying the peak external load in FEA models coupled with a bone remodeling algorithm. Different mechanical stimuli have been investigated, including fluid flow, strain energy density (SED), strain gradient and maximum principal strain, and have shown spatial match of over 47%, less than 9% errors in densitometric parameters in the mouse tail model (Schulte et al., 2013b) and a Kendall’s τ rank coefficient of 0.51 in cortical thickening for the mouse tibia (Pereira et al., 2015). Recently, a combined optimality- and mechanostat-based model using SED as the mechanical stimulus and minimizing the error between the predicted and measured change in geometrical properties between 2 weeks of scans was developed (Cheong et al., 2020a). The results showed that this methodology was able to achieve an overall spatial match of over 60% for healthy, and mechanically loaded tibia after ovariectomy (OVX) (Cheong et al., 2020a, b) at the organ level, and similar bone mineral content (BMC) and bone mineral density (BMD) in healthy or OVX whole tibiae. Physiological loading was applied in those models (Cheong et al., 2020a, b), but the predictive ability of the bone remodeling algorithm under different simulated loading conditions has not been assessed.

The purpose of this study was to evaluate the influence of the organ-level load on the local strain distribution across the length of the mouse tibia, and on the accuracy of the predictions of spatial patterns of bone adaptation, morphometric and densitometric properties spatio-temporally with a multi-scale approach. The analyses were conducted on mouse tibiae that had been mechanically loaded after OVX (Cheong et al., 2020b; Roberts et al., 2020) using physiological load and/or nominal passive load as applied in the experiments. The novelty of this paper is the comparison among predictions of a micro-FE mechanoregulation pipeline driven by physiological load, applied passive load, and combined physiological-passive load as the source of the mechanical stimulus for bone remodeling. In particular, no previous study has investigated the potential influence of physiological loading that the bone is subjected to under daily activities, in addition to the subjected passive load applied during the tibial loading experiments, on bone adaptation and on the predictive ability of the models. We hypothesize that the ability of the multi-scale models in predicting bone changes over time would be similar due to the form-function relationship of bone to withstand loading due to daily physical activities.

## Materials and Methods

### Experimental *in vivo* Data

The experimental data used in this study were collected in a previously published longitudinal study (Roberts et al., 2020). Six 14-week-old female C57BL6/J were ovariectomized at week 14 of age and underwent *in vivo* micro-CT scans of the whole right tibia at weeks 14, 16, 18, 20, and 22 (VivaCT80, Scanco Medical, Bruettisellen, Switzerland) with a scanning procedure (55 kVp, 145 μA, 10.4 μm voxel size, 100 ms integration time, 32 mm field of view, 750 projections/180°, no frame averaging, and 0.5 mm Al filter) that has minimal effects on the bone remodeling (Oliviero et al., 2019). The images were reconstructed by using a beam-hardening polynomial correction based on a 1,200 mgHA/cc wedge phantom (provided by the manufacturer). Mechanical loading was applied to the right tibia *in vivo* at weeks 19 and 21 with a 12N peak load, 40 cycles/day and 3 days/week on alternate days.

### Image Registration

All micro-CT images were rigidly registered to a reference bone following virtual removal of the fibula (Amira 6.3.0, Thermo Fisher Scientific, France), using normalized mutual information as the optimization criterion (Lu et al., 2016, 2017). Following geometrical alignment, a cropping plane perpendicular to the longitudinal axis (z-axis) was used to crop the images to 80% of the tibial length starting from the slice below the proximal growth plate. The gray value histogram of the images was used to compute a global threshold, equidistance between the background and bone peaks, that was used to segment the bone geometry and binarize the cropped images (Oliviero et al., 2017).

### Micro-FE Analysis

**FIGURE 1:**
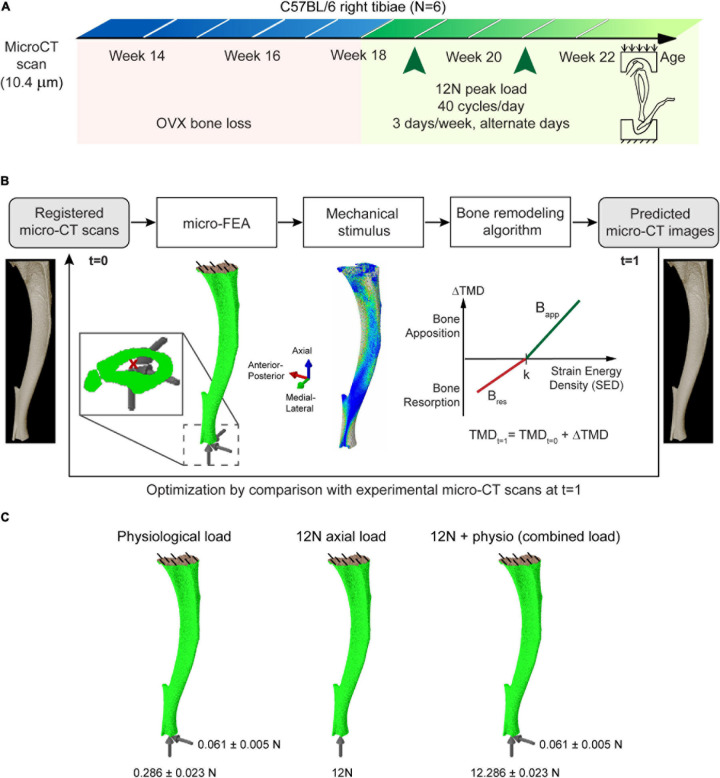
**(A)** Schematic of the *in vivo* loading experiment. **(B)** Overview of the workflow used in determining the parameters of bone adaptation and the computational algorithm used. **(C)** The evaluated loading and boundary conditions.

The segmented images were used to build micro-FE mesh by converting all bone voxels into linear brick elements (element size: 10.4 μm). To determine the changes in structural properties due to changes in bone morphology, tissue homogeneity was assumed (Razi et al., 2015b) and an elastic modulus of 14.8 GPa and Poisson’s ratio of 0.3 was assigned to all element following previous validation studies (Oliviero et al., 2018, 2021a). Micro-FE analysis was performed (Abaqus 2017, Dassault Systèmes Simulia, United States) to obtain the local strain distribution, by applying a load through the centroid of the most distal slice kinematically coupled to the distal surface, while the nodes on the proximal surface were fully constrained (Cheong et al., 2020a). The distal surface was restricted from rotation. This methodology provides a consistent approach to compare changes in the structural properties of bone and has been validated to reproduce the displacement and stiffness under compression using digital volume correlation (DVC) (Oliviero et al., 2018). The images from the treatment period of weeks 18–22 were used for this study and three different types of loading were evaluated: (1) physiological load; (2) nominal 12N axial load; (3) combined nominal 12N axial load superimposed to the physiological load (Figure 1). A peak physiological walking load of 0.01355^∗^BW N/g along the superior-inferior direction and 0.00289^∗^BW N/g along the posterior-anterior direction scaled according to the body weight (BW) of the mouse at each week was applied, calculated using force plate data collected by Charles et al. (2018). The effect of the medial-lateral load was not modeled as its effect on strain energy density was minimal (Cheong et al., 2020a).

### Computational Algorithm and Selection of Parameters

The local mechanical stimulus (strain energy density, SED) was used as input to a linear mechanoregulation algorithm (Figure 1B), applied at the organ-level to predict changes in local tissue mineral density (TMD) as detailed previously (Cheong et al., 2020a). The algorithm is based on Frost’s mechanostat theory, which hypothesizes that bone tissue adapts to the mechanical stimulus by bone formation/resorption until bone is resorbed or the TMD is at equilibrium. Following a sensitivity analysis (Cheong et al., 2020a), a “lazy zone” where net remodeling is zero (Christen et al., 2014) was not implemented as it did not significantly improve the predictions. Gray values of the images were converted to TMD as inputs to generate the predicted micro-CT images at the next time point, using a calibration equation from the manufacturer based on weekly quality checks with a five-rod densitometric phantom. Comparisons of the local SED with the remodeling law was used to compute the mean change in TMD (Cheong et al., 2018), and applied to the background and bone voxels. The updated TMDs were converted back to gray values to generate the pseudo micro-CT images for validation with the experimental dataset. A total of three parameters defined the mechanoregulation algorithm (SED threshold, bone apposition rate and bone resorption rate). The parameters were optimized by computing the change in volumetric second moment in the medial-lateral and anterior-posterior directions between 2 weeks in the binarized experimental and predicted datasets across 10 sections of the bone, and minimizing the difference between the experimental and simulation results using sequential quadratic programming (MATLAB 2018A, The MathWorks Inc., Natick MA, United States).

### Spatial Analyses and Model Accuracy

To identify the sites of bone remodeling, the follow-up images of each mouse were superimposed by aligning their volumetric centroid and cropped to the same length (Cheong et al., 2020b). Surface voxels were determined by locating the endosteal and periosteal outlines of the segmented images. The following densitometric parameters were computed and compared against the *in vivo* dataset, as detailed in Lu et al. (2016): bone volume (BV), bone volume fraction (BV/TV), bone mineral content (BMC) and volumetric bone mineral density (BMD). Bitwise operations were applied to classify increases in TMD on the surface voxels as bone apposition and decreases in TMD as resorption. BV sums the total volume of the binarized bone voxels, while TV sums the volume enclosed by the periosteal surface. BMC for each voxel was computed as the product of the TMD and voxel volume. BMD is obtained by normalizing BMC by TV. To account for measurement uncertainties, the results were computed for the whole bone and averaged across 10 longitudinal sections of the tibia at the tissue level.

Two evaluation metrics were used to determine the accuracy of the model in predicting local bone changes: (1) spatial match, which was defined as the amount of bone changes that has the same state change in the experimental and predicted datasets, normalized by the total number of voxels with the state change in the predicted dataset and (2) prediction accuracy, which was defined as the number of voxels with the same state change in both dataset, normalized by total number of voxels with the state change in the experimental dataset. The prediction accuracy was computed separately for apposition and resorption, and on the endosteal and periosteal surfaces.

### Statistical Analysis

The effect of loading within subjects at each time point was assessed using the non-parametric Wilcoxon signed rank test due to the small sample size. Statistical significance was set at *p* < 0.05 (two-tailed). Heat maps were used to summarize the *p*-values resulting from the comparison of SED in the different sub-regions of the tibia. All data analysis and graph plotting were conducted in Origin 2019 (OriginLab Corp., Northampton, MA, United States).

## Results

**FIGURE 2:**
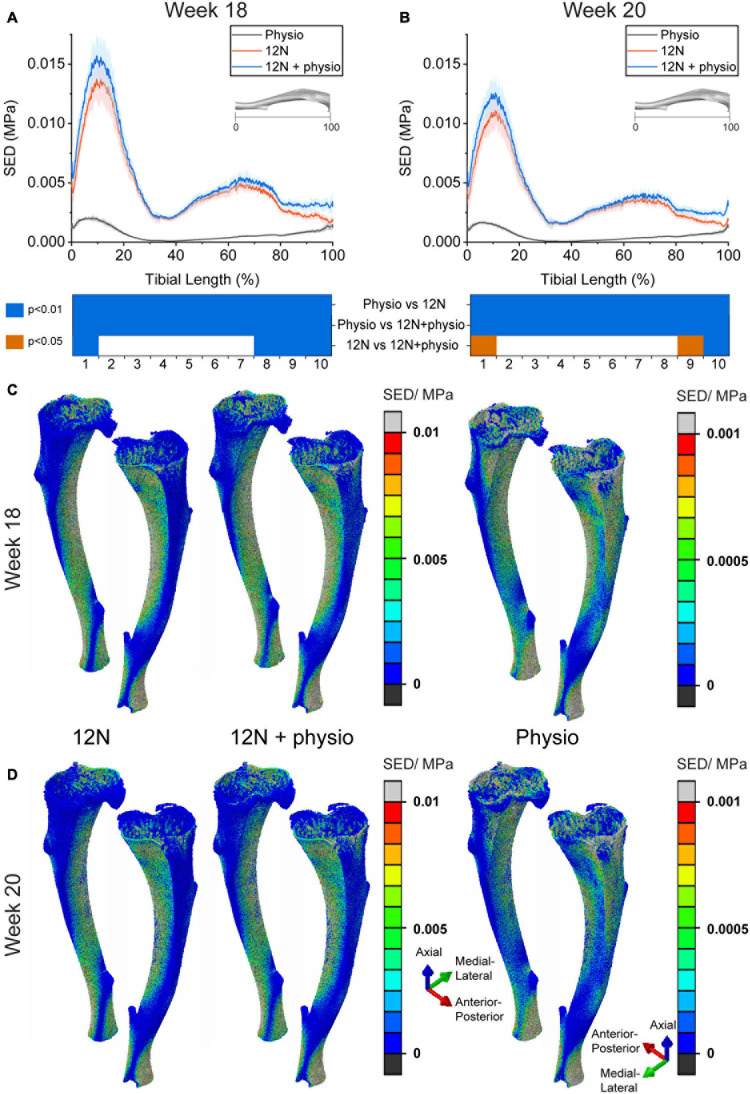
Average strain energy density (SED) in the cross section of the tibia (*N* = 6) due to different loading conditions (Distal: 0%, Proximal: 100%) at **(A)** week 18 and **(B)** week 20. 3D view of SED distribution in a typical mouse tibia (mouse 4) at **(C)** week 18 and **(D)** week 20. Physio: physiological load, 12N: nominal 12N axial load, 12N + physio: combined nominal 12N axial load superimposed on the physiological load. Heat map indicate significant difference between the loading conditions (Wilcoxon signed ranked test; *p* < 0.05: orange; *p* < 0.01: blue). Note the difference in scale for the color maps in the FEA plots.

The results in Figure 2 show the large differences in SED magnitudes and distributions induced in the bone tissue by the passive 12N axial load compared to those induced by the physiological load (physio). At week 18, the SED was significantly different between the 12N load case and the combined case (12N + physio) at the distal (section 1) and proximal tibia (sections 7–10) (Wilcoxon signed ranked test, *p* < 0.05). At week 20, only sections 1, 9, and 10 were significantly different between the 12N and 12N + physio case. Physiological loading caused high SED in both the medial and lateral aspects of the tibia. The 12N axial load causes high SED only on the lateral aspect of the tibia. The posterior region immediately above the distal tibiofibular joint also displayed high SED which was not observed in the physiological loading case. A similar SED distribution was also observed in the 12N + physio loading condition, but with slightly higher SED on the medial tibia.

The boundary conditions simulating the passive loads in the experiments and the combined passive and physiological loads resulted in differences in the SED threshold by 1–2 orders of magnitude compared to the physiological case (Table 1). The SED thresholds between weeks 18–20 were approximately equivalent to 40.5 ± 22.5, 129.0 ± 42.2, and 179.8 ± 82.3 microstrains for the physiological, 12N and combined load case, respectively. At weeks 20–22 the equivalent strain values were 12.8 ± 9.0, 138.9 ± 40.0, and 175.0 ± 66.3 microstrains, respectively. At both weeks 18–20 and weeks 20–22, the SED thresholds were not significantly different for the 12N and 12N + physio load cases (*p* > 0.05), but the apposition rate was different at week 20–22 (*p* = 0.036).

**TABLE 1:** Bone adaptation parameters obtained from optimizing the predicted and experimental images under different loading conditions.

**Time period**	**Load**	**Apposition rate (mg/cc-Pa-2 weeks)**	**Resorption rate (mg/cc-Pa-2 weeks)**	**SED threshold (Pa)**
Week 18–20	Physio	2.1 ± 2.2*	2.1 ± 2.0	15.2 ± 11.9*^#^
	12N	10.6 ± 9.4*	4.0 ± 2.8	134.1 ± 95.3*
	12N + physio	5.8 ± 4.2	2.8 ± 2.2	281.1 ± 270.3^#^
Week 20–22	Physio	2.6 ± 3.0*	0.7 ± 0.2*^#^	1.7 ± 2.1*^#^
	12N	22.5 ± 30.2* ^	6.4 ± 3.9*	152.7 ± 87.7*
	12N + physio	6.8 ± 4.7^	5.9 ± 2.6^#^	253.7 ± 170.9^#^

Physio: physiological load, 12N: nominal 12N axial load, 12N + physio: combined nominal 12N axial load superimposed on the physiological load. *, ^#^, and ^ indicate significant differences between the different groups (Wilcoxon signed rank test: p < 0.05).

**FIGURE 3:**
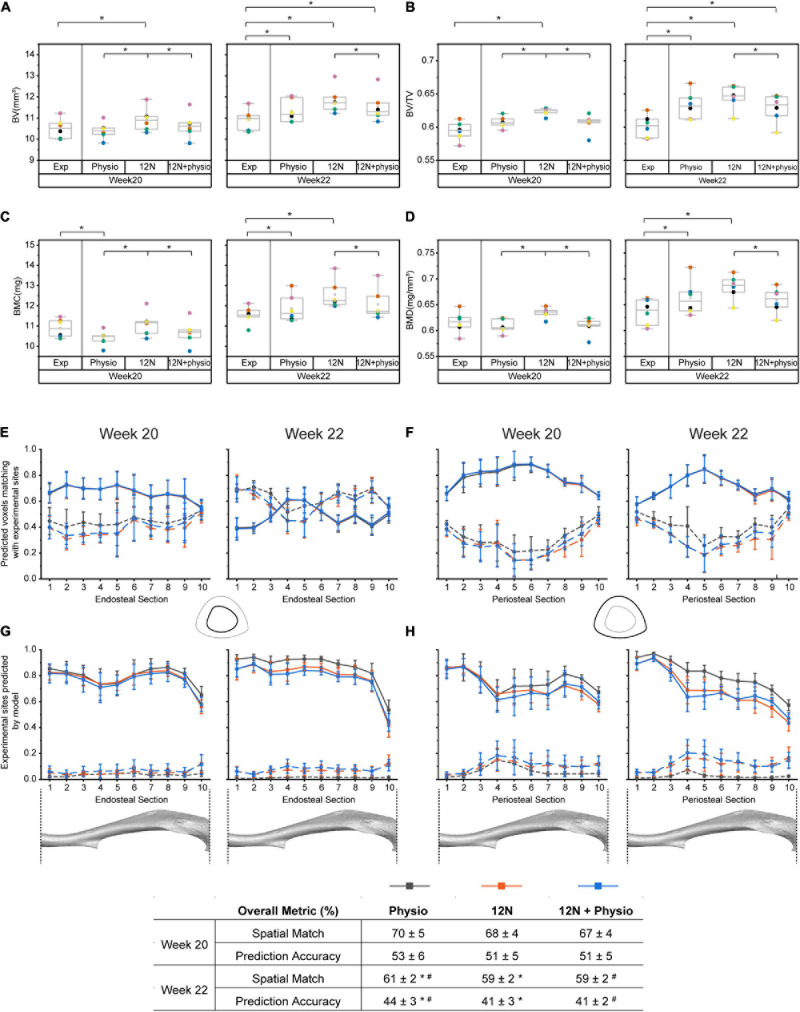
Comparison of the accuracy of different loading conditions on the accuracy of the model prediction. Densitometric parameters were evaluated for: **(A)** Bone volume (BV), **(B)** Bone volume fraction (BV/TV). **(C)** Bone mineral content (BMC), **(D)** Bone mineral density (BMD). The ability of the model to predict the spatial distribution of bone adapation were assessed along the longitudinal axis of the tibia from distal (1) to proximal (10): **(E)** Spatial match on the endosteal surface, **(F)** Spatial match on the periosteal surfaces, **(G)** Prediction accuracy on the endosteal surface, **(H)** Prediction accuracy on the periosteal surfaces. Exp: experimental data, Physio: physiological load, 12N: nominal 12N axial load, 12N + physio: combined nominal 12N axial load superimposed on the physiological load. Solid lines indicate apposition while dashed lines indicate resorption. * and ^#^ indicate significant differences between the different groups (Wilcoxon signed rank test: *p* < 0.05).

Figure 3 shows that the overall densitometric calculations, spatial match and accuracy of the model were similar despite differences in the load magnitudes and distribution in the models. At week 20, the predicted densitometric values were significantly different between the groups, as the 12N axial load condition systematically predicted the highest densitometric values, when compared against the physiological loading (*p* = 0.0036) or combined loading case (*p* = 0.036). At week 22, only the 12N case systematically predicted higher values than the combined loading case (*p* = 0.036). The combined loading condition predicted the closest densitometric values to the experimental data. Regional analysis across 10 sections of the tibia shows that the ability of the combined loading model in predicting the bone changes remains in most cases similar or slightly worse than the predictions with the physiological load alone. At weeks 20–22, the higher apposition and resorption rates in the 12N and 12N + physio case led to a poorer spatial match in resorption, but similar spatial match in apposition compared to the physiological load case (Figure 3).

## Discussion

The overall goal of this study was to assess the effect of the inclusion of physiological loading to the experimental load, in computer simulations of bone adaptation due to *in vivo* axial compression loading. A multi-scale mechanoadaptation model that assumes that local bone adaptation is regulated by the load at the organ level, and determines the parameters of bone remodeling by minimizing the difference in the shape change between the predicted and experimental dataset was used. This is the first time different loading conditions based on nominal passive mechanical loading, physiological loading or a combination of them is implemented. Combined *in vivo* micro-CT and micro-FE analyses were used to assess differences in the strain distribution, the densitometric parameters and local predictions of bone adaptation in the whole bone, and across 10 sections of the tibia.

The SED distributions showed that the induced mechanical stimulus in the proximal and distal tibia were most sensitive to the applied load (Figure 2). The passive axial load applied in the experiments (12N) was approximately 40 times and 200 times larger than the axial and anterior-posterior components of physiological loading, respectively. However, the average SED under 12N axial load was about 6 times higher than when it is under physiological loading, hence demonstrating the large contribution of bending moment induced by the anterior-posterior load. As the strain environment in the mouse tibia is due primarily to bending (Prasad et al., 2010), the absence of anterior-posterior load in the 12N load case may have caused the model to compensate with a higher apposition rate (Table 1). The uncertainties in the boundary and loading conditions have previously been documented at the organ level (Giorgi and Dall’Ara, 2018), but this study shows for the first time the local regions that are most affected by small variations in the passive loading condition. This region-dependence could be due to variations in the second moment of area, which are highest in the proximal 30% of the tibia (Carriero et al., 2018), whereas differences in the distal section of the tibia may be due to the global minimum in cortical area between sections 1 and 2 (Meakin et al., 2017) and its proximity to the boundary conditions.

The strain equivalent threshold (calculated from the SED thresholds) under 12N axial load alone or combined with physiological load was approximately 7–11 times lower than the target peak strain of 1,200–2,000 microstrains (De Souza et al., 2005; Main et al., 2020), measured using strain gauges in mouse tibial loading experiments. This is within the range of 8–12 times difference between the strain equivalent threshold (Cheong et al., 2020a) and the recorded peak tensile strain of <300 microstrain under physiological loading (De Souza et al., 2005). It is difficult to compare the obtained threshold values with literature as previous mouse tibia model utilized poroelastic material properties and SED-based fluid flow to predict locations of bone adaptation under 12N load (Pereira et al., 2015; Carriero et al., 2018). Thresholds of 0.01–0.016 Pa have been used in micro-FE models of the loaded caudal vertebra, selected by matching BV/TV between the predicted and experimental results (Schulte et al., 2013b). Hence the threshold values obtained in this study are considerably lower even after accounting for differences in bone architecture (primarily trabecular bone) and material properties (E = 5.3 GPa, v = 0.3) in their study. Nevertheless, the micro-FE models used in this study have previously been validated for local predictions of displacement using digital volume correlation (DVC) (Oliviero et al., 2018).

The 12N axial loading condition systematically predicted higher apposition rates than when physiological loading conditions were used at both weeks 18–20 and 20–22 (Table 1). This led to better predictions of BMC at week 20, but over-predictions of BV and BV/TV at week 20 and higher overestimation of the densitometric parameters at week 22 compared to the case with physiological loading (Figure 3). The prediction accuracy in apposition was lower at week 22 for the 12N and combined loading conditions. This was due primarily to the stress distribution induced by the 12N axial load concentrating on the lateral regions (Figure 2), even though the experimentally measured apposition was found on both lateral and medial sections (Cheong et al., 2020b). The combined load case improved the predictions of global densitometric properties of the tibia but, surprisingly, without affecting the overall ability of predicting the spatial distribution of TMD changes. The prediction accuracy in resorption for the two cases where the 12N axial load was included were improved, as higher resorption rates were estimated by the algorithm. The significant difference in all densitometric parameters at weeks 20 and 22 for 12N and combined loading shows that the presence of a small anterior-posterior load has a large effect on the minimization of the volumetric second moment in the optimization step.

There are some limitations in this study. Firstly, the growth plate and tibiofibular joint have not been modeled, but sensitivity analysis has shown that the fibula affects the overall bone stiffness and cortical strain distribution (Yang et al., 2014). However, the material properties of the growth plate and soft tissues at the tibiofibular joint are currently not known, and the models could not be validated with the available experimental data. Hence, the focus was on the region of the tibia below the proximal growth plate (Prasad et al., 2010). Homogenous material properties were used in the models to isolate changes in strain distribution due to modifications in shape. The use of heterogeneous material properties would help to understand the role of TMD changes in strain distribution, but would alter the strain profile especially in the trabecular regions (Webster et al., 2012). However, this was not conducted due to the small increment to predictions of structural failure in the murine caudal vertebra (Webster et al., 2012) and mouse tibia (Oliviero et al., 2021b) at the expense of a large computational cost. Although the use of tetrahedral mesh would capture the strain distribution at the surface more smoothly in principle (Cheong et al., 2020a, b), both hexahedral and tetrahedral models of the mouse tibia were found to yield similar results in stiffness, failure load and local strain distributions in the cortical bone (Oliviero et al., 2021b). Moreover, simple loading was applied to quantify the changes to the micromechanical properties, rather than determine the mechanical environment induced by the loading. The influence of the load location in improving predictions of bone remodeling should be explored in further studies. A time period of 2 weeks was used in this study to match with the experimental study. While future work could investigate the use of smaller time steps and to separate the contribution of the 12N axial load and physiological loads, limitations about the number of scans and induced radiation should be considered to maximize the impact of 3Rs (Replacement, Refinement and Reduction of the usage of animals in research; in particular Refinement) in *in vivo* studies (Oliviero et al., 2019). The use of volumetric second moment as the optimization algorithm at the global level biases the algorithm to form bone which resists fracture, and the current algorithm does not optimize changes in TMD. Future work should explore multiple optimization criteria, region-dependency in bone remodeling and inclusion of non-linear mechanoregulation laws. SED was the only mechanical stimulus considered here in line with other studies (Schulte et al., 2013b; Levchuk et al., 2014), as it predicted bone formation better than maximum principal strains (Cheong et al., 2020a). Other stimuli and other mechanisms of bone remodeling, such as micro-damage (Hambli, 2014), fluid flow (Pereira et al., 2015; Carriero et al., 2018), and cell numbers (Lerebours et al., 2016), which have been studied in other models to give realistic predictions in bone adaptation, should also be considered.

In conclusion, predictions of bone adaptation were similar under stimulated physiological loading, external loading, and combined loading despite differences in the SED distribution. This shows that the optimization-based bone adaptation algorithm used in this study, which compares the outputs of multi-scale models combined with longitudinal assessment of bone changes over time in the mouse tibia, is primarily driven by changes in the geometrical form of the bone. The combined 12N axial and physiological loading conditions marginally improved the densitometric predictions compared to physiological loading alone. Thus, the results show that the form-function relationship of bone is due to both the 12N axial load and daily physiological load. The similarity in the ratio of peak strain to the SED threshold for simulated loading and simulated physiological loading (without passive loading), suggests that part of bone’s response to applied load, specifically the SED threshold, can be estimated from the induced peak strains in the midshaft under different passive loads. However, the apposition and resorption rates will be linked to the presence of tibia positioning and other experimental uncertainties.

## Data Availability Statement

The datasets analyzed for this study can be found in Figshare (https://doi.org/10.15131/shef.data.12927365).

## Ethics Statement

The animal study was reviewed and approved by the Research Ethics Committee of the University of Sheffield.

## Author Contributions

VC and ED designed the study. VC planned and conducted the computational modeling, analyzed the data, and drafted the manuscript. All authors discussed the results, reviewed and commented on the manuscript.

## Conflict of Interest

The authors declare that the research was conducted in the absence of any commercial or financial relationships that could be construed as a potential conflict of interest.
